# Antibiotic Resistance and the Biology of History

**DOI:** 10.1177/1357034X14561341

**Published:** 2015-03-13

**Authors:** Hannah Landecker

**Affiliations:** University of California

**Keywords:** antibiotic resistance, antibiotics, biology, biomedicine, biopolitics, biotechnology, social studies of science

## Abstract

Beginning in the 1940s, mass production of antibiotics involved the industrial-scale growth of microorganisms to harvest their metabolic products. Unfortunately, the use of antibiotics selects for resistance at answering scale. The turn to the study of antibiotic resistance in microbiology and medicine is examined, focusing on the realization that individual therapies targeted at single pathogens in individual bodies are environmental events affecting bacterial evolution far beyond bodies. In turning to biological manifestations of antibiotic use, sciences fathom material outcomes of their own previous concepts. Archival work with stored soil and clinical samples produces a record described here as ‘the biology of history’: the physical registration of human history in bacterial life. This account thus foregrounds the importance of understanding both the materiality of history and the historicity of matter in theories and concepts of life today.

A recent American Centers for Disease Control threat report on bacterial pathogens refractory to treatment with antibiotic therapies contains a blunt warning: ‘simply using antibiotics creates resistance’ (CDC, 2013: 14). Solutions have become problems, putting biopower out of joint. Measures and places of biological control, hygiene or bodily discipline teem with antibiotic resistant bacteria. Pets, supermarket meat, hospital drains, locker rooms, and lungs, guts and sores harbour or suffer antibiotic resistance. Antibiotic resistance is a collective ecological condition of late industrialism (Fortun, 2012; Orzech and Nichter, 2008). It appears at once in these intended targets of therapeutic control, and increasingly off-target – in urban crows, plants, coastal waters, beached whales, lice, soil, aquarium fish – as the bodily condition bleeds into the environmental condition (Oravcova et al., 2013; Rose et al., 2013).

Antibiotic resistance is a topic proper to epidemiologists, microbiologists and physicians; and to health economists and public health officials focused on the practical problem of *what to do* in the face of crisis. At the same time, antibiotic resistance presents a critical problem of *what to think* in and beyond biomedical science. Antibiotics kill by selective toxicity, disrupting microbial structures or processes that do not exist in human cells. Their production is driven by theories of *antibiosis*: a human leveraging of substances microbes create in mutually antagonistic battles for space and resources. Humans make antibiotics by farming microbes, chemically tinkering with microbial metabolites, and mimicking them with synthetic antibiotics. Antibiotic resistance arises when microbes gain the capacity to evade these drugs.

Rethinking antibiosis – anti-life – in this moment of its crisis is a problem that challenges historical and cultural theories of events and bodies as much as it does microbiological or evolutionary theory. In framing antibiotic resistance as a problem for cultural critical theory of life, health and the body, this article has three parts, which build on one another. The first section elaborates the history of wars and experiments in which antibiotics, produced by microbes, became an industrialized biological (Landecker, 1999). This emphasizes the scale of manufacture of antibiotics, which themselves quickly became infrastructural to the production of many other things at scale: more health, more meat, more fruit, more surgery, less death, more fertility, in everything from *in vitro* embryos cultured in antibiotics to fish farming. The scale of production is also the scale of resistance.

Second, scientific theories of antibiotic resistance are traced from prior logics of mutation – that assumed bacteria to be simple single-celled individuals – to recent theories of horizontal gene transfer. This shift from mutation to gene flow models is a realization of the trans-individual effects of an environment structured by the industrial scale of antibiotic manufacture described in the first section. Through tools for tracking horizontal gene transfer, scientists have come to see the historical record of human antibiotic use inscribed across the biology of bacteria: to see individually applied therapies meant only to act on the diseases in the bodies of sick people or animals as historical environmental events that drive the evolution of pathogens and commensal bacteria alike, in bodies and far beyond them. This turn to horizontal flow from vertical inheritance has profound practical and conceptual consequences for notions of bodily integrity, immunity and individuality, both medically and culturally (Blackman, 2010).

Third, these first two sections of the article are used to build a concept of the *biology of history*: how human historical events and processes have materialized as biological events and processes and ecologies. The history involved is the specific 20th-century intersection of sections one and two: where industrial scale meets concepts of bacteriological control and individuality. Using the tools of ethnography and history of science, this analysis focuses on medicine and microbiology’s study of the biological processes by which their own former knowledge configurations and technical practices have changed bacteria. A historical record is being traced through archival work in stored clinical and soil samples; what is seen is antibiotic use materialized as shifts in the mode and tempo of bacterial evolution.

In the history of biology, ideas of bacteria change. In the biology of history, the bacteria of ideas change. The bacteria of today are not the bacteria of yesterday, whether that change is registered culturally, genetically, physiologically, ecologically or medically. Bacteria today have different plasmids and traits and interrelations and capacities and distributions and temporalities than bacteria before modern antibiotics. It is not even clear that ‘bacteria’ remains the only or the most salient category with which to think about antibiotic resistance. This biological matter, chewing away its own ontology, is historically and culturally – and materially – specific to late industrialism, produced in and by previous modes of knowledge.

## Methods and Contributions

This article analyses scenes of microbiology and medicine as they now contend with large-scale biological effects of their own previous knowledge formations. Practically speaking, I have focused on accounts of the material (genetic, evolutionary, physiological or ecological) manifestations of past actions or assumptions. These are selected from a voluminous historical and contemporary literature on antimicrobial resistance, my interviews with laboratory and medical scientists, and observations of scientific conferences. It is not possible to step outside of the frame – outside of the language of science and technology – for direct access to the materiality of life as it changes. There is no getting out of this particular petri dish. Rather, I attend critically to 21st-century life sciences as they grapple with their own biotechnical legacies, analysing this moment of involution.

This account joins a growing literature in the social, philosophical and cultural study of science invested in microbial life as a site for making ‘theory out of science’ (Paxson and Helmreich, 2013; Roosth and Schrader, 2013), as well as an expanding scholarly corpus on materiality and vitality in the human sciences (Dolphijn and van der Tuin, 2012; Fraser et al., 2006). It departs from this literature by focusing on the materiality of history and the historicity of matter undergirding theoretical change in science and beyond. The 20th-century logic of control and the mass enactment of it on humans, animals, microbes and landscapes *has* a history, and simultaneously *is* a history – an unfolding of some parts of life and the disappearance or suppression of other parts (cf. Malabou, 2008: 1).

Antibiotic resistance ruptures assumptions about divisions between human history (of culture, politics, discourse and science) and natural history (of genes, evolution, ecological relationships, population size and distribution, physiological and reproductive processes): assumptions that would allow that the science of bacteria could have historicity, but bacterial matter could not (Heidegger, 2009; Koselleck, 2002; Chakrabarty, 2009). Antibiotic resistance confronts history of science and theories of conceptual change with a double movement in which the science of biology changes – but so does the biology of science, driven by the industrialization of bacterial metabolism. It is common to hear: ‘we used to think … but now we know’, as knowledge shifts; such reaching into the unknown and constantly correcting the course of knowledge is constitutive to the dynamics of scientific practice (Rheinberger, 2010). We used to think a certain way – about ether, or genes – then ideas changed as more became known.

In the case of antibiotic resistance, we might rather say: ‘We used to think a certain way about antibiosis and pathogens. And then we changed the future.’ What we thought we knew became the biology under study: the solution has become the problem. Not all sciences confront the contours of their past logics as mass irruptions at global scale of thoroughgoing changes in forms of life. Bacterial life today is appearing as a specific instantiation of the biology of the Anthropocene: human efforts to control life’s productivity become the matter of the world. It is a deeply anthropocentric narrative about humans and their thoughts and actions, while at the same time the consequences of antibiotic resistance are forcing a decentring of the very units of analysis that we might use to decide what is human, non-human, animal, viral, species, bacterial, embodied, environmental, intentional, or engineered in the first place. Let us begin on familiar territory – with the history of humans and their actions – and strike out for less certain ground from there.

## Antibiotics at Scale

Antibiotics were a constitutive element of a triumphal period of 20th-century medicine (Bud, 2007). Penicillin, produced by the *Penicillium* mould, was named by Alexander Fleming in 1928, who noticed its ability to inhibit bacteria in culture dishes. Penicillin was developed as a drug by Norman Heatley, Ernst Chain and Howard Florey in wartime England. It effectively treated bacterial infections in mice and, shortly thereafter, people; it cured previously untreatable diseases, and its greater efficacy and relatively fewer side effects than therapeutic agents such as sulfonamides made it appear a ‘miracle drug’. Today, however, few research articles or reviews recount the triumphal narrative; instead, they draw attention to scale. ‘The amount of antibiotics produced since the beginning of the antibiotic era in 1950 is obviously very considerable, and one wonders if it may be significantly more than what is produced naturally in the biosphere, given that antibiotics are made in barely detectable amounts in soil’ (Davies, 2006: 287).

A bare 70 years after the discovery of a fungal inhibitor of bacterial growth, production of antibiotic molecules reached millions of metric tonnes a year.

### Penicillin and the Second World War

Meticulous work by historians of biotechnology reveals the conjuncture of pre-Second World War biotechnologies of microbial fermentation with the wartime drive for penicillin to treat armies in the United States (Bud, 2007; Neushul, 1993). Florey and Heatley came to the US in 1941 seeking help with penicillin production; this resulted in collaboration with workers at the United States Department of Agriculture (USDA). Moulds were already being grown in large vat fermenters to harvest the gluconic, oxalic and citric acids that were microbial metabolic products. Citric acid was (and is) mass produced for the American market; used to flavour soft drinks in high demand during prohibition, it also preserved food’s colour and flavour in the canning process (Bud, 1994).^1^ The British request for help with penicillin thus entered into a scene in which moulds such as *Aspergillus* were already cultured en masse. Pfizer, a company that entered the pharmaceutical market with the advent of penicillin, previously worked only in food products; it began producing citric acid with *Aspergillus niger* in 1919, spurred on by the disruption of Italian citrus exports during the Frist World War.

In a remarkable hybridization of local biologies with mass production, the task of producing penicillin came to the USDA’s Northern Regional Research Laboratory (NRRL) in Peoria, Illinois. To find high-yield strains of *Penicillium*, the American Army Transport Command sent soil samples from around the world, but the most productive strain came from a mouldy cantaloupe purchased in Peoria. For growth medium, instead of the brewer’s yeast solutions the British had used, the Americans developed corn steep liquor by soaking corn kernels in water, cheaply garnered from the Midwest fields. Experience gained from culturing acids for food production and consumer goods such as cleansers was brought to bear on penicillin: 10,000-gallon vat fermenters, complete with internal means to agitate the contents, ensured the mould could grow throughout the medium not just on the surface (Neushul, 1993). The term *fermentation* refers to metabolic processes in which microbes and their enzymes transform corn sugars into acids, gases and alcohols to be used in industry or therapy. Vat fermenters optimized the conditions for the resident organisms’ conversion of food source into metabolic products valued by humans.

As the United States entered the war, its War Production Board was instrumental in taking these vat fermentation techniques and high-yield strains to industrial pharmaceutical producers such as Pfizer, Merck and Squibb. Combined efforts of government agencies, university research scientists and industry enabled rapid scaling up. At first, soldiers’ urine was collected to recycle ingested penicillin. By 1944 enough to treat armies was produced in factories (Bud, 2007: 23). In 1945, it was released to the general public in the US, and production was estimated at 270 lb a month – adequate for 9 million people a month (Sokoloff, 1945: 47). In wartime, penicillin was depicted as saviour of the battle-injured – but it was equally or more important in maintaining army manpower diminished by venereal disease (Neushul, 1998).

### Medicine Transformed

Diseases that previously could not be stopped and catastrophic bacterial infections from suppurating wounds were abruptly and seemingly magically cleared up. In 1941, just before the introduction of penicillin, the mortality rate from *Staphylococcus aureus* infections that had reached the blood stream was reported to be 80% (Skinner and Keefer, 1941). Before mid-century, pneumonia was the leading cause of death in America; after, it was not. Early stories of antibiotic cures focused on miraculous cures of individuals: a postman rescued from sepsis, the housewife resurrected from streptococcus though near unto death (Sokoloff, 1945). In concert with the development of a vaccine for polio and therapeutics such as corticosteroids for arthritis, antibiotics transformed medicine and mortality rates. Penicillin for the civilian population was a matter both of disease and reproduction: congenital syphilis caused miscarriage and birth defects; gonorrhoea also yielded to penicillin treatment rapidly. By 1950 in the United States, the major classes of prescription domestic drug sales were estimated by industry analysts at: hormones, $100m; sulfonamides $150m; vitamins $200m; and antibiotics $250m. International *exports* of penicillin were valued at $46,392,000 by the Federal Drug Administration (FDA), and other countries were busy setting up their own production facilities on the American model (Raper, 1952).

### Beyond Penicillin: Soil Bio-prospecting

Commentators remarked that antibiotics were surpassing the value of the other fermentation-based industry: alcohol. The search was on for more such substances: ‘Whereas scientists once had nothing but oaths for the lowly mold that would alight on their agar plates, [today] a mold-contaminated plate is treated like a king’ (Sokoloff, 1945: 98). Real riches didn’t come from the lab, but from the soil. ‘Equal to its importance as a drug,’ wrote the president of the American Mycological Society of America, ‘has been [Penicillin’s] effect of precipitating and sustaining the unprecedented search for other drugs of microbial origin. Everywhere the searchers say: “If it can happen once, surely it can happen again”’ (Raper, 1952: 15). And it did, over and over again. Just one such discovery story is enough to illustrate this mid-century soil bio-prospecting.

In 1943, Lederle Laboratories, a division of the heavy industrial chemical business American Cyanamid, hired Benjamin Duggar, a retired professor of economic botany, to search for new microbes producing non-toxic drugs (Nelson and Projan, 2005). He was an expert in soil fungi and their use in economic activity; he had developed widely adopted methods for the mass production of cultivated mushrooms (Duggar, 1905). He was 71 in 1943 and, in the search for new antibiotics, leveraged a large and geographically dispersed network of colleagues in soil science built over a career, asking them to send dirt from undisturbed sites. One sample, dug from a dormant hay field in Missouri, yielded an actinomycete bacterium that Duggar named *Streptomyces aureofaciens*. Fermentation of the organism produced the antibiotic Aureomycin, approved by the FDA in 1948 and patented in 1949, becoming the drug of choice to treat typhoid fever and other previously untreatable bacterial ailments. Again, the intensely local – the soil culture of a particular corner of the earth – was scaled up and widely distributed.

Lederle scientists discovered by accident that the waste products of antibiotic production could promote growth in animals, and quickly entered an already thriving market in feed supplements (Stokstad et al., 1949).^2^ They sold antibiotics to farmers on the premise that tiny amounts added to animal diets produced ‘dramatically faster growth, less disease, and earlier marketability’ in chickens, turkeys, cows and pigs, ‘without detectable loss in meat quality’ (Raper, 1952: 34). Even the mink farmers, it was reported, were ecstatic with this inexpensive addition to their animals’ diets, producing 20% larger pelts (Ratcliff, 1951). The combination of medical and agricultural uses has only grown in scope. Though precise numbers are hard to come by, ballpark ones convey the scale: in the 2000s, the United States had reached production numbers in the area of 50 million lbs per year, and there alone, a billion lbs of antibiotics have likely been produced already (Davies, 2006: 287).

Medical history was therefore also environmental history. Within ten years of isolation, penicillin production was global in scale and scope, spurring prospecting and cultivation of other soil microorganisms. The metabolisms of microbes were joined to those of animals and humans in new ways, at scale. Animals were brought inside from outdoor cultivation and fed antibiotics.^3^ They grew to market size on the same amount of food in less time, contributing to post-war increases in meat consumption (Jukes et al., 1950).

At the same time, antibiotics changed medicine, moving from treatment to prophylaxis, externally applied with creams and lozenges, and ingested with increasing regularity. Industrial microbiology provided the capital foundation for the biotechnology industry emerging in the 1970s, as companies turned from producing biologicals such as vitamins and antibiotics to more exotic recombinant products (Rabinow, 1996). Although the development of new antibiotics has declined precipitously since the 1960s, manufacture of existing ones has continued apace. Today millions of tonnes of antibiotics are produced in this way around the world.

## From Mutation to Horizontal Gene Transfer

A constant shadow on this story of biotic control has been the appearance of resistance: bacteria once killed by antibiotics suddenly oblivious to them. Hannah Arendt wrote of it in *The Human Condition*:

Modern motorization would appear like a process of biological mutation in which human bodies gradually begin to be covered by shells of steel. For the watcher from the universe, this mutation would be no more or less mysterious than the mutation which now goes on before our eyes in those small living organisms which we fought with antibiotics and which mysteriously have developed new strains to resist us. (1958: 323)

Already in 1958, antibiotic resistance seemed part of the modern human condition. Penicillin was given to the first patients in 1941. Penicillin resistant bacteria were detected 1942. And so it goes: Methicillin introduced 1960. Methicillin resistance reported 1961.

### The Logic of Mutation

Used in the laboratory to select for mutants, antibiotics became a valuable research tool of genetic science. It was assumed that antibiotics selected a few resistant mutant individuals from a population, particularly if a low dose was applied (to a human or a culture dish). Few survived but these went on to multiply and reconstitute the population. In the clinic, the solution for antibiotic resistance was to seek another antibiotic to avoid the mutation. An outbreak of penicillin resistant *Staphylococcus aureus* began in hospitals in the UK in 1942; the synthesis of penicillinase-resistant penicillins followed (Rammelkamp and Maxon, 1942). Early on, penicillin was noted as a wonderful treatment for sulfonamide-resistant pneumonia (Sokoloff, 1945: 77). A review of the literature of antibiotic resistance appeared in 1948 (Bailey and Cavallito, 1948).

Antibiotic resistance was recognized as a problem and yet seemed not to be an urgent one. Complacency prevailed: another drug could always be found, existing drugs could be further altered, and it was assumed to be an infrequent problem affecting non-compliant patients. It was thought that mutation events would be rare, remaining limited to a mutant’s descendants, channelled by vertical genetic inheritance. Thus resistance was experienced as a spur to discovery of drugs working by the same principle, not as a fundamental challenge to the model.

### Infective Heredity

A marked shift in theories of bacterial evolution came with Joshua Lederberg’s 1950s elaboration of alternate modes of genetic transmission between individual bacteria (Brock, 1990). ‘Transduction’ named the process by which bacteriophage, the viruses that infect bacteria, can carry bits of bacterial DNA with them as they travel between bacterial hosts. Lederberg also studied the biology of the ‘plasmid’– a small extra-chromosomal circle of DNA that moves between bacteria during conjugation. These entities, he theorized, formed a spectrum of ‘infective heredity’ in which bacteria could catch genes, even across species (Creager, 2007: 179). In addition to transduction and plasmids, mobile genetic elements such as transposons were found to encode the enzymatic means of their own excision from and reinsertion into DNA, ‘jumping’ onto chromosomes or plasmids, again within or between bacteria.

At first, these facets of infective heredity remained a focus of molecular biology, as unique properties of bacteria that could be exploited experimentally. Plasmids were used to move DNA in and out of cells, a practice that led to genetic engineering and recombinant DNA biotechnology (Hughes, 2001). It might seem coincidental that a key enzyme used in the development of genetic engineering – a restriction enzyme that would snip DNA sequences with targeted precision – came from an antibiotic resistant bacterial strain isolated from a patient (Creager, 2007). However, multiple histories can simultaneously unspool in the same room, operating at different rates. In this case, the intentional engineering of bacterial genomes has been the thread that critical social science scholarship has followed. The story has been humans making life, or at least remaking it to their own ends and modelled on their own desires – nature intentionally modelled on culture (Rabinow, 1992; Giddens, 1991).

Increasingly visible, however, is another story moving at a different pace: the unintentional widespread mobilization of mobile DNA bringing new genetic features into chromosomes and plasmids and driving global antibiotic resistance. Who was engineering what in the 20th century? What looked then like a laboratory technique ready to remake the world can also be retold as a remade world about to remake the laboratory. In the same years as the ascendance of genetic engineering, the advent of multiple drug resistant bacterial illnesses made plasmids and other mobile DNA elements the focus of medical microbiology. From here unfolded a very different understanding of resistance and bacterial genomes; plasmids in these medical settings looked less like clean technologies of control and more like imminent disasters.

### Plasmids as Historical Composites

In 1959, faced with the anomaly of patients with resistance to multiple antibiotics that they had never been exposed to, Lederberg’s work on transduction and plasmids suddenly seemed relevant to medicine. In Japan, researchers investigating a multiply drug resistant *Shigella* bacterial infection in a patient showed that these traits could not have arisen by four separate mutation events in the same bacterium. They identified what they called an ‘R-factor’ (resistance factor) that moved between bacteria (Creager, 2007).^4^

Only when multiple drug resistant bacteria began crowding microscope slides and clinical samples did infectious disease researchers begin to take further notice. The ‘R-factor’ finding, obscure when it was first published, soon sounded like a warning knell for similar phenomena seen at epidemic proportions. Mass outbreaks of antibiotic resistant bacterial dysentery swept Central America in 1969, making hundreds of thousands sick and killing thousands; the infectious agent was a bacterial species thought to have been eradicated by antibiotics, but which suddenly re-emerged multiply drug resistant (Farrar, 1985).

The tools of molecular biology were turned on the plasmids in the *Shigella* bacteria crossing nations. A history of antibiotic application was seen layered into the plasmid’s constitution. ‘The resistance patterns exhibited by these organisms have included those antibiotics that were being used most heavily at the time of the outbreak, as well as older agents, and the resistance markers were usually on one or more plasmids’ (Farrar, 1985: 1103). In other words, plasmids seemed to collect different resistances, including to antibiotics no longer in widespread use. Again it provided an explanation of how one patient with one infection could have resistance to many antibiotics, regardless of whether they had ever personally been treated with them; but instead of a few patients, there were thousands.

The prevalence of antibiotic resistance in different countries layered according to the history of the introduction of antibiotics to their markets: in Mexico, multi-drug resistant *Shigella* were most commonly resistant to the sulfonamides introduced in the 1940s, followed, in descending frequency of incidence, by resistance to penicillin, chloramphenical and tetracycline, which were introduced later and in that order (Levy, 2002). Thus it was realized that the plasmids, as well as other mobile bits of DNA called transposons and integrons, carry multiple resistance genes and pick up more over time.

### Epidemic Plasmids

Once scientists started following plasmids carrying antibiotic resistance markers instead of pathogenic bacteria, they realized that these genetic pieces did not stay contained in species. When gentamicin was introduced in the 1970s, an intercontinental, cross-genera, cross-species spread of resistance to that antibiotic’s specific mode of action was observed, due to the spread of an ‘epidemic plasmid’ (O’Brien et al., 1985).^5^ Studying antibiotic resistance therefore drove a shift in perspective. Rather than studying infectious diseases one at a time (dysentery, pneumonia, etc.), this was the study of infectious heredity among microbes, in which genes encoding resistance and virulence traits spread via horizontal gene transfer. Use of one antibiotic targeted at one kind of bacteria resulted in multiple resistances across many species of bacteria; mutation and vertical inheritance could not explain this phenomenon.

The ability to sequence large amounts of DNA very quickly, along with the bioinformatics tools to bank and compare sequences with each other, sharpened the sense of genomes as historical composites formed across species. Horizontal transfer occurs between individual cells in the moment – within the time of a single generation – but what is transferred may be a pastiche of other slowly accrued, vertically inherited features. For example, a plasmid isolated from the human pathogen *Corynebacterium striatum* had a mosaic structure ‘comprising eight DNA segments the boundaries of which are represented by horizontal mobile elements’: these eight segments came from ‘bacteria of different habitats and geographical origin … that last shared a common ancestor about 2 billion years ago’ (Tauch and Pühler, 2002: 29). This plasmid’s multifarious constitution came together within the last 50 years, amalgamating long spans of evolutionary history under the short sharp pressure of antibiotic exposure: assemblage, *par excellence*.

### The Bacterial Pangenome

Mutation in its general sense means alteration. It also means change in the structure of a gene resulting in a variant form inherited by subsequent generations. In this neo-Darwinian, gene-oriented sense, mutation is receding as the explanation for bacterial change under antibiotic pressure, inadequate to the task of describing the non-individual, multiple-gene phenomenon described above. Now we see a shift from ‘mutation’ to words like the ‘pangenome’ of bacteria (Gillings, 2013). ‘Indeed the exchange of genes is so pervasive that the entire bacterial world can be thought of as one huge multicellular organism in which cells exchange their genes with ease’, writes Stuart Levy (1998: 48), a scientist long active in calling political attention to antibiotic resistance. This pan-organism has a pangenome.

The subset of the pangenome that encodes resistance has been dubbed ‘the resistome’ (D’Costa et al., 2006); it is understood as a common pool from which all bacteria, pathogenic or benign, native to soil or to animal, aerobic or anaerobic, can potentially draw on under the selective pressure of antibiotics. Research on resistance focuses on the novel acquisition, by a formerly susceptible organism, of the ability to survive antibiotic exposure. A resistant bacterium can cut the antibiotic molecule into pieces, chemically alter the antibiotic or its target, or pump the antibiotic out of the cell – or sometimes all of those things and more. To be resistant, a bacterium must have a gene that codes for the means of resistance, for example an enzyme that cuts antibiotics, or a protein efflux pump.

Following the trail of genes that code for resistance-enabling functions, scientists have realized that plasmids and other mobile genetic elements are the rule not the exception in bacterial life. The horizontal mode has come to be appreciated as the major source of genetic change in bacteria over time (Helmreich, 2003; O’Malley and Dupré, 2007). Horizontal transmission ‘potentially makes all genes in the microbial biosphere a single, common, and shared resource’ within which bacteria can ‘mobilize and transfer genes across physical and phylogenetic distance very rapidly’ (Stokes and Gillings, 2011: 790).

### The Example of ‘Iraqibacter’

Horizontal gene transfer and the vertical mode of reproduction within a species are not opposed, but are intersecting modes of proliferation over space and time; while the bacterium is reproducing and spreading between hosts such as lice and people, it is ‘catching’ new DNA. The survival and reproduction of the mobile genetic element is tied up with that of the bacterial host. New DNA is kept and reproduced through cell division if it aids in the survival and proliferation of both the genetic mobile element and the bacterium.

An example helps illustrate this intersection. ‘Iraqibacter’ or *Acinetobacter baumannii* is a bacterial species that was a benign soil inhabitant but became an antibiotic resistant pathogen starting around 1963. It has troubled military hospitals since the first Gulf War, and by 2008 ‘*A. baumannii* strains resistant to all known antibiotics have now been reported, signifying a sentinel event that should be acted upon promptly by the international health care community’ (Peleg et al., 2008: 539). How could it change so quickly?

One clinical isolate of *A. baumannii* harboured a ‘resistance island’ containing 45 resistance genes derived from several other species of bacteria picked up in a single horizontal transfer event (Fournier et al., 2006). The percentage of recently acquired genes in *A. baumannii* is currently estimated at 17%, including genes from *Legionella pneumophilia*, identified in 1976 when attendees of an American Legion convention fell ill with serious lung infections (Smith et al., 2007). The bacterium travels a variety of miserable conduits, gaining antibiotic resistance and virulence along the way; in 2004, an *A. baumannii* epidemic among human body lice was identified, found in lice collected from around the world.

### Individual Therapies as Environmental Events

Drug resistant pathogens such as *A. baumannii*, methicillin resistant *Staphylococcus aureus* (MRSA), or, more particularly, infectious agents of tuberculosis and gonorrhoea capture attention because they make people ill and are difficult or impossible to cure. However, the implications of horizontal gene transfer are not confined to either pathogens or humans. It seems likely that ‘most resistance determinants persist and amplify not in clinical isolates’ but in commensal ‘reservoirs’; that is, not in human disease-causing bacteria but in the commensal bacteria that normally live in humans and animals (Levy and Marshall, 2013). There are more multi-drug resistant pathogens because of change in frequency and distribution of resistance genes across bacteria *in general*.

Antibiotics have global effects far beyond their intended targets in part because resistance genes move around together in clusters. The American domestic bee population, for example, treated for decades with tetracyclines, has a gut microbiome harbouring resistance to tetracycline – *and* ampicillin, not used in bee husbandry. In Europe antibiotics are not used in beekeeping and these resistance patterns are not seen (Tian et al., 2012). Treating an individual with a single antibiotic generates a selective pressure whose effects are general; pathogenic or commensal bacteria in or around the treated individual often become resistant to many antibiotics other than the one used in treatment.

An antibiotic meant as an individual intervention is an environmental event, with effects spilling out beyond the target body (or hive). Antibiotics have been called a ‘societal drug’: when one person in a household takes an antibiotic for an extended period, for example to treat acne, density of antibiotic resistant bacteria on the skin of everyone else in the household increases (Levy, 1998). In day care units and hospitals, where antibiotic use is high, individuals carry higher loads of antibiotic resistant bacteria even if they are not themselves being treated with antibiotics. Individual human bodies and the institutions they occupy are sinks through which the bacterial pangenome can flow.

These ecological ramifications of a single targeted use are also due to the physical persistence of the antibiotics in the environments around treated individuals. Antibiotics do not disappear after being ingested; a portion of those consumed by humans and animals are excreted from bodies into wastewater, alongside bacteria and genetic elements carrying resistance traits: ‘cells and genes are disseminated simultaneously with the original selective agents via human waste streams’ (Gillings and Stokes, 2012: 346). Selection for resistance by low, sub-lethal concentrations of antibiotics in soil and water happens all around bodies, not just in bodies directly treated therapeutically (Baquero et al., 2013). Antibiotic resistance becomes the latent condition of the environment through which pathogens travel and are more likely to become (more) antibiotic resistant (Murphy, 2011).

### Horizontal Gene Transfer and Mass Production

Antibiotics and antibiotic resistance came together and changed together. All of this talk of plasmids and transposons and transduction and genomes can make the environmental component of the story recede. But the concept of selective pressure stitches the first part of this article to the second: the scale of manufacture and use becomes the scale of selection for resistance. This is not a question of this pathogen or that bacterium, of particular medical uses or agricultural applications. Population-level interventions with antibiotic molecules for decades become population-level reservoirs of resistance genes in human and animal and plant and insect commensal bacteria. Antibiotic resistant bacteria are usually harmless and go undetected; antibiotic resistance genes alone do not cause pathogenicity. Organisms today carry high loads of antibiotic resistance determinants in their microbiota – this is not illness, but an evolved condition of bacterial populations exposed to antibiotics in increasing amounts since 1950 (Sommer et al., 2009).

In human population-scale studies of commensal bacterial populations (populations of populations), the kind and frequency of antibiotic resistance found in the human microbiome correlates with antibiotic usage in medicine and agriculture in given countries (Forslund et al., 2014). Comparison of ‘contemporary’ gut bacteria to archived samples from 1972 and 1982 from healthy individuals and animals shows that ‘application of antibiotics has caused an increase in the incidence of resistance to these antimicrobial compounds, even within bacterial species that are *not directly subject* to antibiotic control’ (Houndt and Ochman, 2000: 5407, emphasis added).

Antibiotic resistant human disease is everywhere, but far from uniform, because of the variegation in kinds and amounts of antibiotics used in medicine and public health and agriculture. Paul Farmer and his collaborators argue that World Health Organization efforts in the 1990s to make first-line antibiotics cheap and short-course chemotherapy for tuberculosis readily available, coupled with World Bank policies aimed at decentralizing health care, created an ecology of sub-optimal short doses of first-line antibiotics. Thus drug resistant tuberculosis proliferated, particularly in nations such as Peru (Kim et al., 2005). The effect of any intervention, in which antibiotics are viewed as specific to certain pathogenic targets, is lateral, potentially multiple and latent; a route by which social management of disease becomes infectively heritable through populations. Large-scale shifts in microbial being accompany plans for the management of the health of populations.

## The Biology of History

In the quote that opened the previous section, Hannah Arendt wrote that modern motorization was akin to ‘a process of biological mutation in which human bodies gradually begin to be covered by shells of steel’, which was like the mutations by which bacteria came to resist antibiotics (1958: 323). Revisiting this history from the viewpoint of contemporary research in microbial metagenomics makes these parallels seem less metaphorical than literal – motorization and industrialization become modern biology. The mechanically agitated, corn-fed, X-ray and UV-mutated, radically augmented metabolisms and expanded lives of *Streptomyces* and *Penicillium*, packaged, marketed and distributed en masse, became the ecology and the biology of bacteria.

It is a biology of the industrialization of global nutrient flows that hugely magnifies some metabolic capacities and feeds them onward, reshaping genomic time and space. Today the corn liquor of mid-century has been replaced by soy broth mixed with blood meal, cottonseed flour, pork liver or herring meal, and other ‘unlikely nutrients for soil microbes’ (Yim et al., 2006: 164). It is difficult to say what the watcher from the universe might make of feeding animals and plants to microbes in order to feed antibiotics to animals and plants in order to feed humans who are themselves (relatively speaking) doused in antibiotics. Mutation hardly seems adequate to the task of describing these historical developments.

Thinking from the perspective of horizontal gene transfer rather than mutation demonstrates that antibiotic resistance is not an infectious disease *of* humans. It is a phenomenon of infectious heredity among microbes, in which genetic material encoding resistance and virulence traits spreads between microbial cells and species in environments in which antibiotics are present. Having explored the history of antibiotic production, and the conceptual changes driven by confronting genetic changes in human pathogens, this article now turns from the history of biology to the biology of history.

In its simplest form, this means the biological imprint of human actions, a form of ‘xenogenetic pollution’ in which DNA elements have ‘fixed in populations, largely as a result of human use of selective agents’ (Gillings and Stokes, 2012: 346). However, nothing to do with antibiotic resistance is simple. In its more complicated form, the biology of history refers to a recursive structure in which knowledge is produced in and through matter that itself has been altered by previous modes of thought (Franklin, 2013). At the same time that we now know more, we come to inhabit the material future produced by what we thought we knew.

In what follows, I analyse the turn to archival work on the part of microbiology and medicine, using stored clinical and soil samples to fathom the effects of previous theories and the interventions they supported (Salyers et al., 2004). In this work, bacteria are far more than a kind of historical record analogous to words or pictures. Microbiologists are fathoming the way modes of thought at the basis of their own discipline have driven biological change (Knapp et al., 2009). The effects of presuppositions are material, such that the very thing under study has the human history of explanation and intervention within it.

### About Finding Yesterday’s Theories to Be in Error

Of particular note in the current literature of antibiotic resistance is reflexivity about the production of resistance showing up erroneous assumptions of bacterial simplicity and individuality. For example:

Bacteria are highly social organisms that communicate via signaling molecules, move collectively over surfaces and make biofilm communities. Nonetheless, our main line of defense against pathogenic bacteria consists of antibiotics – drugs that target individual-level traits and thus, regrettably, select for resistance against their own action. (Boyle et al., 2013: 207)

At the same time, antibiotics, long assumed to be weapons by which one species of microbe killed or inhibited another, are rethought as ‘intermicrobial signaling agents’ within microbial community dynamics (Linares et al., 2006). Because they are found in ‘sub-inhibitory’ concentrations in soil, it has been proposed that most ‘antibiotic’ compounds are means of communication and gene regulation in and between bacterial species. It is humans that use them as weapons (Yim et al., 2007). If antibiotic molecules are actually not things that kill, but things that communicate and coerce, their use has been at deafening concentrations for many years now.

Recognition of past errors is a characteristic aspect of science. Historian-philosophers of science from Gaston Bachelard, through Georges Canguilhem and Hans-Jörg Rheinberger, have identified a recursive structure to scientific endeavour, in which ‘new knowledge arises that constantly challenges [us] to rethink the presuppositions of the method in use’ (Rheinberger, 2010: 22). This recursive structure produces *constitutive historicity* for sciences always looking to see how the truth of today will become an error of the past, constantly being driven beyond the current configuration. And yet, by understanding its course to be a corrective of previous presuppositions, it remains constantly ‘recursively related’ to the past from which it is busy departing (Rheinberger, 2010: 22). In other words, it is not unusual for microbiology to find past assumptions erroneous; in fact it is constitutive of the dynamics of knowledge.

However, nature does not change out from under most sciences. Moreover, even if things under study change, they are understood to do so with their own temporalities: they are accorded *natural* history. Antibiotic resistance is both a special case and an instructive departure specific to the nexus of scientific knowledge and industrialization of biological process. Theories of the recursive structure of science have not had to take account of knowledge changing the material world at such a scale. The example of antibiotic resistance stitches the philosophy of science to its sociology, its economics, its mass production: ‘give me a laboratory, and I will raise the world’ (Latour, 1983, 1993) is now followed on by give me a world (transformed en masse by antibiotics), and I will raise a laboratory (in which the scientific question has become antibiotic resistance).

### From the Archive: ‘Ancient’ Resistance

Did humans cause antibiotic resistance? The answer scientists are formulating, through the elaboration of various pasts become present, is both no and yes. These answers return us to the question of biological control at scale. On the one hand, no: neither antibiotics nor antibiotic resistance are of human origin. Humans got antibiotics from microbes. Most clinical antibiotics are originally derived from soil bacteria; not surprisingly, those bacteria ‘harbor resistance elements for self-protection’ and ‘genes orthologous to these have been identified on mobile genetic elements in resistant pathogens in clinical settings’ (D’Costa et al., 2006: 374).^6^

Thus antibiotics and antibiotic resistance may come from the same source: soil bacteria harbour both the capacity to make antibiotics and the capacity to live with antibiotics. Moreover, these capacities are ‘ancient’: to prove it, samples of ‘ancient DNA from 30,000 year old Beringian permafrosts’ were taken from the Yukon tundra, and there indeed were antibiotic resistance determinants similar to modern forms (D’Costa et al., 2011). Some soil microorganisms can subsist on antibiotics as their sole carbon source (Dantas et al., 2008). To ‘eat’ an antibiotic, a bacterium must be capable of degrading it: ‘catabolic pathways responsible for antibiotic digestion in nature provide a rich source of potential resistance determinants’ (Davies and Davies, 2010: 423). Antibiotic resistance can arise and spread so easily because it is already there.

### The Soil in People

On the other hand, the human role in antibiotic resistance is evident: just because it already existed doesn’t mean that it had the scale, mode and tempo that it does now. Take the example of Aureomycin, discussed above. *Streptomyces aureofaciens* was harvested from a rural corner of Missouri, and brought into a life of mass production in 1943. Half of the antibiotics used clinically today are from species within this single genus, *Streptomyces*. While humans and animals have had a relationship with soil for the span of evolutionary history, humans for the most part have not lived within the soil. The story of the excavation of the earth, the mass production of soil bacteria in order to extract the riches of their antibiotic molecules, is simultaneously the story of bringing the dynamics of the soil world up into the air. Bringing, in a certain sense, augmented soil ecologies into humans, through an infective heredity brought on by sheer physical juxtaposition: smearing it on the skin, putting it in surgical incisions, giving it to babies to eat, feeding it to animals that humans eat. Much like the mushrooms for which Duggar developed methods of commercial cultivation, millions of tons of antibiotics are ingested every year. Brought up from the earth with these bacteria are ecological dynamics that drive genome evolution and resistance.

### Mode and Tempo

The frequency and distribution of antibiotic resistance is an ecological event driven and sustained by the scale of antibiotic usage that began in the 20th century. The bacteria that live in and on humans and animals – the many species that have evolved to live in niches such as cow rumens and human noses – have historically been minimally antibiotic resistant. They didn’t live in a soil ecology where companions and competitors were making antibiotics. Even if they were exposed to soil dynamics, these molecules were at such low concentrations that they didn’t need antibiotic resistance traits to simply survive; they do now. Again, science returns to the scene of its past; testing of clinical specimens from the first decades of the 20th century shows that modern human pathogens and commensal bacteria possessed as many plasmids as they do now, but those plasmids contained antibiotic resistance at very low frequencies (Hughes and Datta, 1983).

Historical exploration has led to the hypothesis that the mode and tempo of bacterial evolution have changed through antibiotic use. Prompted by hostile environmental conditions of antibiotic application, some bacteria increase their release and uptake of genetic material, as well as increasing genetic recombination at chromosomal sites as a stress response (Guerin et al., 2009). If bacteria survive to outcompete others that lack antibiotic resistance, the volume and spatial dissemination of resistance increases. This is why the pattern of usage of antibiotics is quickly reflected in bacterial genomes: their presence accelerates genetic exchange or gene duplication itself. At the same time, other aspects of industrialization may be driving antibiotic resistance indirectly; exposure to heavy metals or disinfectants can drive the same gene exchange processes in soil bacteria (Gillings and Stokes, 2012).

Environmental pressure may also select for bacteria – such as *A. baumannii* – that are more ‘porous’ to genetic flow. The acquisition of foreign DNA is not necessarily advantageous, and bacteria have protective mechanisms for degrading invasive transposons and bacterial virus DNA. Strong selective pressure to keep invasive elements tips the balance toward less ‘protective’ bacteria: ‘it is highly probable that the general tempo of lateral transfer has actually increased due to selection on cells with inherently higher rates of lateral transfer’ (Stokes and Gillings, 2011: 801).

This accelerated tempo is visible through archival work: ‘contemporary bacteria’ show a much denser clustering of resistance and virulence genes than those in archived soil or clinical samples, showing that the ‘ancient’ mobile genetic units have new mosaic structure evolved under antibiotic pressure (Gillings and Stokes, 2012). Scientists hypothesize that human-generated pressure on mobile elements to retain resistance and virulence determinants is changing the kind and cargo of these elements. If they make the bacteria more likely to survive, mobile elements proliferate. And when mobile elements with more resistance genes are more frequent in the environment, it is more likely that pathogens will acquire them.

### The Unintended Biology of Biotechnological Control

The mechanisms of genetic mobility in bacteria have been there for a very long time, well before humans; but they never before carried such cargo as they do today. Antibiotic molecules and antibiotic-degrading capacities are also ancient; but they never existed in ‘nature’ in the kinds of concentrations seen after 1950. Our commensals, our pathogens, our parasites, our domestic animals and fish and their commensals, the pathogens of our parasites, the avian scavengers of our cities and the wildlife – are all now participating in an antibiotic ecology.

Understanding that antibiotics came originally from soil bacteria, that soil bacteria exist in ecologies in which antibiotics are part of competition for space to live in and cooperative metabolic interactions, that human/animal commensal bacteria have not historically possessed the resistance mechanisms that they are now acquiring under the pressures of the scale of application of antibiotics – all this brings into focus a view of the antibiotic era as a massive rearrangement of an existing biology, which will never again be as it was. *Pneumococcus* recedes, *Staphyloccocus* ascends. *Helicobacter pylori* is wiped out and the incidence of ulcers declines; obesity rises (Blaser and Falkow, 2009). The number of plasmids carrying resistance genes increases in the microbiosphere and the rate of ‘mutation, recombination, transposition, modularization, gene transfer’ goes up (Baquero et al., 2013: 9).

Tracings of biotechnological control in microbiological matter are visible in other ways. Widespread childhood vaccination against *Streptococcus pneumoniae* (isolated by Louis Pasteur as the infectious agent of pneumonia in 1881) is a public health success story, drastically reducing pneumonia incidence. Simultaneously, a population of increasingly methicillin resistant *Staphylococcus aureus* (MRSA) has expanded. *S. pneumoniae* were competitors with *S. aureus* in an unrecognized ecological relationship. The traditional practice of ‘pure culture’, in which one strain of bacteria is cultured alone in a dish in the laboratory at a time, did not predict that successful suppression of one would make room for the other (Blaser and Falkow, 2009). Training populations of human immune systems to recognize and destroy *S. pneumoniae* was supposed to expand the possibilities for human life by reducing morbidity and mortality. It worked. And it also amplified the possibilities and potentialities of other life.

More than the *discourse* of antibiotic resistance has changed since earlier calls to pay attention to this phenomenon (Cooper, 2008). More than the meaning attributed to bacterial genes has changed, more than the *framing* of threat and risk (Lee and Motzkau, 2013). It is not only that an increasing epidemiological frequency of high-profile scary outbreaks of bacteria such as MRSA has been matched by the fervour with which they are publicly enumerated (McKenna, 2010). There is a concurrent material shift in the numbers, kinds, temporalities and capabilities of bacteria and genetic elements; there is a shift in the rate of exchange and the ensuing survival and proliferation of some life as well as the suppression and extinction of other life.

## Conclusion

In this story, we have seen that lice can have epidemics of bacterial infection; bacteria have epidemics of plasmid infection; plasmids have epidemics of transposon and integron infection. Our epidemics have epidemics; our populations have populations. Celia Lowe, drawing on microbiology’s narratives of highly mutable viruses, has named such phenomena ‘multispecies clouds’, to evoke both the kinds of organisms brought together and the ‘clusters of biosocialities’ mediated by the viral quasi-entity (Lowe, 2010). Yet even species begins to look like a rather tenuous way to piece the quilt. From the perspective of horizontal genetic fluidity ‘what an organism is, and whether something is part of an organism or not, are not questions that necessarily admit of definitive answers’ (Dupré, 2012: 153). Thinking alongside these shifts in scientific theory requires a perspective on biology that is not contained in different organisms – as though cell membranes and skins and so on were little packages for life that then engage in transactions (Hird, 2007).

In the post-vital 1990s, it seemed that DNA sequence information would become the object of life science, leaving behind the bodies of living beings (Doyle, 1997). However, the collection of vast amounts of gene sequence data has instead led to a realization of the frequency and prevalence of horizontal gene transfer and thus its centrality in the constitution of bacterial genomes. Rather than post-vital, this kind of meta-genomic work is a post-organismic vitalism, one that holds little regard for the individualization of life into discrete pieces, whether those pieces are individual bacteria or the species taxonomies humans built for bacteria in the late 19th and 20th centuries (Sapp, 2009; Helmreich, 2009). The scale of the material shift in the post-organismic, post-species bacterial pangenome – and the startling ubiquity of antibiotic resistance that it has produced – becomes comprehensible as the particular biology of modern history.

The story, of course, does not end there; life goes on. What we might call a biopolitical rationality of antibiotic resistance – rather than of antibiotic control of infectious disease – really began to emerge in the 1990s. Concerted activism by scientists such as Stuart Levy, who founded the non-profit group Alliance for the Prudent Use of Antibiotics in 1981, rallied and organized scientific and political action and public alarm (White et al., 2005). Paul Farmer, a physician-anthropologist who has laboured for decades to bring the world’s attention to the phenomenon of multi-drug resistant tuberculosis, has also played a key role in capturing global political attention (Farmer, 1999). The phenomenon of antibiotic resistance is now being framed in no less than apocalyptic terms (Smith and Coast, 2013).

After Foucault (2008), we tend to think of biopolitical rationalities as having and enacting specific knowledge regimes. However, we must also consider the biology of history – the biology of 20th-century biopolitics – in interaction with political change. At this intersection we find the emergence of political rationalities of antibiotic resistance today (Collier and Lakoff, 2014). Biopolitics in the 21st century is increasingly forced to face up to the unexpected material growths of yesterday’s techniques of control, which were enacted at national or global population scale: that is, with understanding and dealing with the biological wages of 20th-century technological optimism yoked to the manufacturing revolution.

This framework could extend to other 20th-century technologies of control of health and reproduction, such as synthetic hormones. These too began to be industrially produced in the 20th century; these too became growth promoters in agriculture in the 1940s; these too are now manifesting as unanticipated biologies at scale (Langston, 2010). Antibiotics and hormonal mimics are not just analogous, but completely intertwined at a material level, from antibacterial soaps with endocrine-disrupting properties, to the novel micro-environments housing bacteria in fragments of plastics floating in the ocean, to the interactions of xenobiotic hormone-like molecules and bacteria in the animal microbiome (Snedeker and Hay, 2013; Zettler et al., 2013). As with antibiotics, the task of managing vitality turns to the control of the substances that were the previous technologies of production: regulating endocrine disruptors, regulating antibiotic use. It is a strange time of controlling prior modes of control.

The analysis provided here has focused on an account of a biology of history made legible by contemporary microbiology’s encounter with the material forms of its prior assumptions. This involution represents a shift in the conditions for producing knowledge about the relationship of the 21st century to its recent past, concerned as it is with the historicity of microbial and genetic matter. Also implicated are social and cultural analyses of biomedicine and health, as this case pushes us to see that concepts and tools, and economics and politics of life are changing, but so are biologies. Our theories and empirical work should speak to the complex materiality of life adapting to management and manipulation at enormous scale well beyond the frame of human intention. The history of antibiotics is not behind us, it is in us. Such is the nature of life today.

## Notes

1. World production of citric acid was estimated at 1.6 million tonnes in 2007 (Berovic and Legisa, 2007).

2. Vitamin B_12_ had recently been isolated and identified as a substitute for the so-called ‘animal protein factor’ – a dietary factor essential to growth for animals fed non-variegated diets of alfalfa, corn or soy meal. It was known that bacteria made vitamin B_12_. After antibiotic molecules were harvested, thousands of gallons of bacterial sludge had to be discarded; it was reasoned that perhaps the waste products of antibiotic production could supply animals with B_12_. What was unanticipated, but quickly pursued, was that this bacterial ‘mash’ spurred animal growth more than could be accounted for by B_12_ alone; even the trace amounts of antibiotics left in the sludge promoted growth.

3. At the same time, animals began to suffer from vitamin deficiencies caused by living on concrete floors (which reduced their exposure to soil microbes) and having their native microbiota suppressed by antibiotics. These deficiencies were fixed by feeding the animals vitamins harvested from yet more microbes, part of a strong market in biotechnologically produced B vitamins.

4. The history of research into horizontal gene transfer is beyond the scope of this article. In addition to the medical and the molecular biology traditions mentioned here, research on horizontal gene transfer was independently pursued in the agricultural-botanical domain: it was demonstrated that genetic material in *Agrobacterium tumefaciens*, the bacteria that cause crown gall in plants, was integrated into infected plant cell chromosomes in the late 1960s, proving DNA transfer between kingdoms. Another genealogy belongs to the world of molecular phylogeny, comparing sequence data between organisms to try to figure out their evolutionary relationships (Helmreich, 2003). See Syvanen and Kado (2002) for examples of all these research fields, and their recent synthesis into a single topic called horizontal gene transfer.

5. Single point mutations in bacterial genes – changes to a single nucleotide in the DNA sequence – also track the history of drug innovation. The bacteria that became resistant to penicillin early on generated an enzyme that broke the chemical structure of the penicillin molecule. In response, scientists began to tinker with the drug’s structure, and brought methicillin to market in 1960. By 1961, however, methicillin resistance had appeared, and then a ‘steady evolution of beta-lactamases (enzymes that detoxify the beta-lactam antibiotics) by point mutation under the selective pressure of successive introductions of new beta-lactamase resistant penicillins, cephalosporins, carbapenems and monobactams’ (Davies, 2006: 289). A single nucleotide substitution mutation could necessitate ‘the development of a new antibiotic derivative at the cost of tens of millions of dollars’ (Davies, 2006: 289). These mutations, once acquired, were rapidly transmitted and amplified through bacterial populations through horizontal gene transfer.

6. One study isolated and cultured 480 strains of soil bacteria with 21 antibiotics, including natural products, their semisynthetic derivatives, and completely synthetic molecules (representing the span of types of antibiotics used clinically). All sampled bacteria were multi-drug resistant; on average each bacterium was resistant to 7–8 antibiotics. ‘There were no antibiotics for which a resistant organism was not found’; it was the ‘default phenotype’ of soil bacteria (Wright, 2007: 180).
